# High efficiency laser resonance ionization of plutonium

**DOI:** 10.1038/s41598-021-01886-z

**Published:** 2021-12-06

**Authors:** Alfredo Galindo-Uribarri, Yuan Liu, Elisa Romero Romero, Daniel W. Stracener

**Affiliations:** 1 Physics Division, Oak Ridge National Laboratory, Oak Ridge, TN 37831 USA; 2 Department of Physics and Astronomy, University of Tennessee, Knoxville, TN 37996 USA; 3 Present Address: Facility for Rare Isotope Beams, Michigan State University, East Lansing, MI 48824 USA; 4 Present Address: Helmholtz-Institut Mainz, Johannes Gutenberg Universität Mainz, 55128 Mainz, Germany

**Keywords:** Planetary science, Optics and photonics, Physics

## Abstract

Three-step resonance photoionization spectra of plutonium have been studied with Ti:Sapphire lasers for the development of efficient laser ionization schemes for ultra-trace analysis of Pu isotopes by resonance ionization mass spectrometry. We observed eighteen intermediate excited states of even parity in the energy range 35568–36701 ${\text {cm}}^{-1}$, thirteen of them have not been previously documented, and a larger number of high-lying excited states and autoionizing states of odd-parity between 48238 and 49510 ${\text {cm}}^{-1}$. Three-color, three-photon ionization schemes via six intermediate states were evaluated under similar ion source operating conditions. This led to a highly efficient three-step scheme with an overall ionization efficiency of $51.1 \pm 1.3\%$, which is an order of magnitude improvement over the previously reported ionization efficiency for Pu.

## Introduction

Plutonium (Pu) is one of the most important actinide members for its use in nuclear weapons and nuclear energy and its ubiquitous presence in the Earth’s surface environment as a result of anthropogenic activities, such as nuclear weapons tests, accidents at nuclear power plants, release from nuclear fuel reprocessing facilities, poor disposal of nuclear wastes, and loss of nuclear weapons^1–3^. Although the amount of Pu in the environment is small, it is considered a highly hazardous pollutant due to its high radiotoxicity and potential risk to human health. Pu could also be naturally produced, like all the heavy elements making up the Earth, by rapid neutron-capture process (r-process) in stellar nucleosynthesis^4^. The observation of ultra-trace $^{244}$Pu, the longest-lived Pu isotope with a half-life of about 80 million years, in deep-sea crust and sediment is indicative of possible stellar nucleosynthesis sites such as neutron star mergers^5^. Hence, detection of Pu concentration and the abundance ratios of its various isotopes, in particular $^{238-242,244}$Pu, in environmental samples and nuclear materials has vitally important applications in nuclear forensics and safeguards^6^, environmental science^3^, radiation protection at nuclear facilities^7^, and cosmochemistry and astrophysics^5,8^. However, the Pu amount in the samples is usually at very low trace levels. The most sensitive analytical techniques are required for these applications.

Resonance ionization mass spectrometry (RIMS) is one of the most sensitive techniques for ultra-trace analysis of long-lived radionuclides such as the isotopes of actinides^9–14^. In RIMS, ions are formed by stepwise resonant absorption of two or three photons through allowed atomic levels to photoionization. The multistep resonance process is extremely selective for the specific element of interest. Thus, in comparison with the commonly used mass spectrometry techniques^15^, such as accelerator mass spectrometry (AMS), inductively coupled plasma mass spectrometry (ICP-MS), and thermal ionization mass spectrometry (TIMS), RIMS is superior in suppressing the isobaric interferences in the subsequent mass separation and promises high detection efficiencies^10,12,16^ and thus high sensitivities^10,17,18^. In this paper, we present the studies of efficient resonance ionization schemes for ultra-trace analysis of Pu isotopes by RIMS.

Trace detection of Pu isotopes by RIMS was first demonstrated by Donohue at al.^19,20^ and Krönert, et al.^21^. In these early studies, Pu atoms were ionized by single-color, one or two-photon excitations. Three-color, three-step resonant ionization was first reported by Peuser et al.^22^ using three pulsed dye lasers, which showed a detection efficiency of 10$^{-7}$. Since then, there have been extensive studies on RIMS as an analytical instrument for trace analysis of Pu and other radioactive elements^17,23–31^, for fundamental studies of actinides^32^, and in combination with other techniques such as SIMS^30,32,33^, fast chemical separation^29^, and laser ablation^34^. Worden et al.^35^ studied two- and three-step resonance ionization pathways using dye lasers. Grüning et al.^26^ and Kunz et al.^27^ investigated two-color and three-color, three-step ionization schemes using tunable solid-state Ti:Sapphire (Ti:Sa) lasers. Due to these efforts, the RIMS detection limit has been substantially improved to $10^4 - 10^5$ Pu atoms^17^ and work is underway towards the detection limit of 1000 atoms for $^{244}$Pu in interstellar dust grains^18^.

In spite of the prior work, an efficient ionization scheme for Pu is still lacking. To our knowledge, the best overall efficiency for Pu is 5% by in-source RIMS^17^ and 5% ion yield with a RIMS instrument^18^. Pu has the atomic ground-state electronic configuration of [Rn]5f$^6$7s$^2$ with a partially occupied 5f-shell and open 6d-shell. Like other actinides, it has complicated spectra with high density of energy levels due to many possible electronic configurations and strong configuration interactions^36^. On the other hand, the large number of bound and autoionizing (AI) electronic states could offer pathways for more efficient photoionization. Recent studies have shown highly efficient resonance ionization of actinides^16,37^ and lanthanides^38,39^. Therefore, using all of this prior work as a foundation, we conducted spectroscopy surveys of intermediate, high-lying, and AI states in Pu, in order to identify suitable multi-step ionization pathways for Ti:Sa lasers, and evaluated different three-step schemes under similar experimental conditions^10^. As a result, a three-step scheme is demonstrated with an overall ionization efficiency of over 50%. The experimental procedure and results are described.

## Experimental

**Figure 1:**
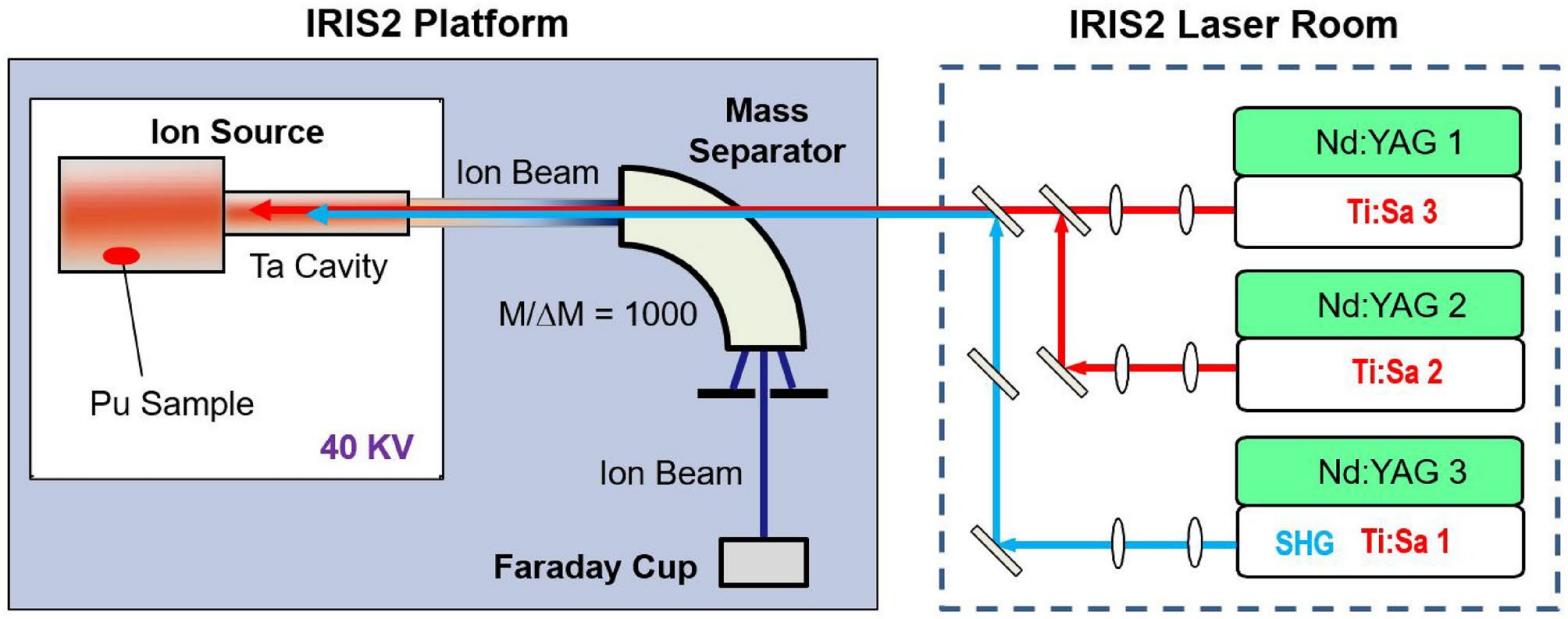
Schematic view of the experimental setup.

The experiment was conducted with the hot-cavity resonance ionization laser ion source (RILIS) and the Injector for Radioactive Ion Species 2 (IRIS2) mass separator^40^ at the Oak Ridge National Laboratory (ORNL). The experimental setup is illustrated in Fig. 1 and the detailed components have been previously described^10,41,42^. Hence a brief description is given here.

The RILIS consisted of a hot-cavity ion source and a system of three Ti:Sapphire (Ti:Sa) lasers. The ion source had a Ta tubular cavity of 3 mm inner diameter (ID), 1 mm wall, and 30 mm in length, which was connected to a closed-end Ta sample tube of 8.5 mm ID and about 100 mm in length that served as a crucible for the sample. The ion source assembly was resistively heated to high temperatures. With a heating current up to 450 A, the cavity could be heated to temperatures exceeding 2300 K while the sample tube could reach 1800-2000 K. Pu atoms were produced by evaporating a Pu sample inside the sample tube. The evaporated atoms effused into the cavity where they were irradiated by laser beams which were tuned to selectively ionize Pu atoms. The ions were extracted from the cavity, accelerated to 40 keV, and transported to a magnetic mass separator with a nominal $\frac{m}{\Delta m}$= 1000 mass resolving power for mass number A = 100. All Pu isotopes could be ionized. The mass separator was optimized for transmission of the A = 242 ion beam. The $^{242}$Pu ion beam intensity passing through the mass separator was measured with a Faraday cup (FC). All the FCs in the experimental setup were secondary-electron suppressed with a repelling voltage of $-300$ V. The laser beams were delivered into the hot Ta cavity through a quartz window on the “straight-through” port in the vacuum chamber of the mass separator magnet, traveling in the opposite direction of the ion beam. The three laser beams were collimated and merged into a single laser beam inside the laser room, which was then transported to IRIS2 and focused over a distance of more than 10 meters into the 3-mm cavity of the ion source. The laser beam size entering the hot-cavity was chosen to be slightly larger than the cavity ID (3 mm) in order to fill the cavity for high ionization efficiency. The typical efficiency of laser transportation into the cavity was measured to be 70–80% after the beams were merged. Accordingly, the full width at half-maximum (FWHM) of the laser beams in the laser-atom interaction region was estimated to be 2.4–3 mm.

### Ionization scheme

**Figure 2:**
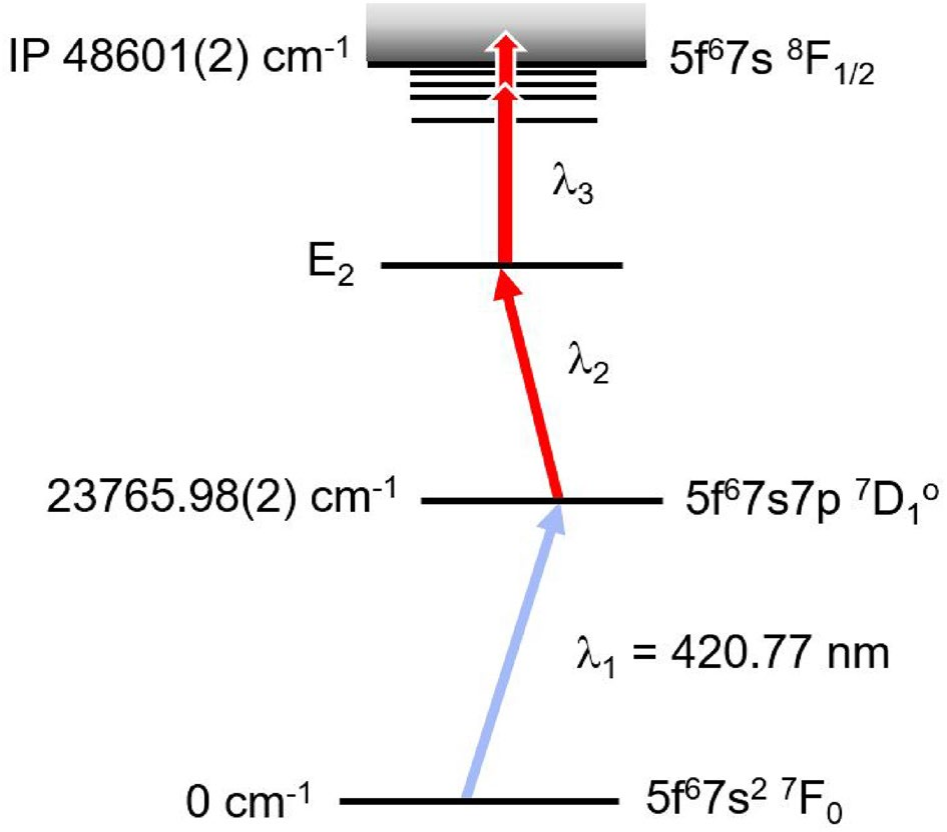
Three-step ionization scheme for $^{242}$Pu investigated. The first excited state energy 23765.98(2) ${\text {cm}}^{-1}$ is from Ref.^26^ and the IP value is from the NIST database^43^. The laser wavelength is in vacuum.

Three-color, three-step resonant excitation of Pu was investigated (Fig. 2). The first step was a known transition^26^ from the 5f$^6$7s$^2$ $^7F_0$ ground state to the first excited state 5f$^6$7s7p $^7D_1^\circ$ at 23765.98(2) ${\text {cm}}^{-1}$ for $^{242}$Pu induced by the first laser ($\lambda _1$ = 420.77 nm). The excited Pu atoms were subsequently excited by the second laser ($\lambda _2$) to the second excited state (SES) from where they were ionized by absorbing another photon from the third laser ($\lambda _3$). The three laser beams were provided by a system^10,42^ of three pulsed Ti:Sa lasers tunable between 720 nm and 960 nm. All the wavelengths stated in this paper are given in vacuum. The first photon at 402.77 nm was produced by second harmonic generation (SHG) of the fundamental radiation of the Ti:Sa laser. The second and third steps employed the Ti:Sa laser fundamental output. The typical laser power in this study was 200 mW for the first step and 1.5 W for the second and third steps. The three laser beams were individually collimated and then merged into a single laser beam. The merged laser beam was focused into the 3-mm cavity of the ion source with an average injection efficiency of 60-70%. The Ti:Sa lasers were individually pumped by three Q-switched Nd:YAG lasers at 532 nm at 10-kHz pulse repetition rate. The pulse lengths of the Ti:Sa lasers were on the order of 30 ns. The three laser outputs were synchronized in time to better than 5 ns via synchronizing the individual pump lasers.

The experiment was conducted by first scanning the wavelength of the second laser $\lambda _2$ to search for available intermediate excited states E$_2$. Then, with $\lambda _2$ tuned to a specific wavelength to populate a selected E$_2$, the third laser wavelength $\lambda _3$ was scanned to obtain three-step photoionization spectra. In all cases, the first excitation step was the same. The fundamental wavelengths of the three Ti:Sa lasers were measured simultaneously with a calibrated wavelength meter (HighFinesse WS6-600) equipped with a 4-channel opto-mechanical switcher. The absolute accuracy of the wavelength meter was 0.02 ${\text {cm}}^{-1}$ according to a 3-sigma criterion, which was taken as the systematic uncertainty in measuring the laser wavelengths.

### Pu sample preparation

The Pu samples were prepared from Certified Reference Material for $^{242}$Pu (CRM 130, plutonium-242 spike assay and isotopic standard) purchased from New Brunswick Laboratory (NBL). The CRM 130 contained 4.1403 ± 0.003 $\upmu$moles of $^{242}$Pu in the form of evaporated plutonium nitrate with 99.94623 ± 0.00065 % purity and traces of other Pu isotopes, each below 0.025%. The isotopic standard is designed to prepare a master solution having a known concentration of plutonium on a weight basis. The sample and the 30 ml Teflon container were handled under strict radiologically controlled conditions in a dedicated hot laboratory. The standard was first dissolved in 1 ml of 8 M nitric acid solution at 100 $^\circ$C, which was then diluted to 10 ml with de-ionized water to a final concentration of 100 ppm $^{242}$Pu. Different sizes of Pu samples were prepared with different amounts of the 100 ppm $^{242}$Pu master solution. For spectroscopy studies, the sample was made with 400 $\upmu$l of the 100 ppm $^{242}$Pu solution containing a total of about 10$^{17}$ $^{242}$Pu atoms. The sample solution was dried on a thin zirconium (Zr) metal foil of approximately 8x8 mm size and then wrapped in the foil. At elevated temperatures, the plutonium nitrate was expected to reduce to atomic Pu using Zr as the metallic reductant. In this study, laser ionized $^{242}$Pu ions were observed at sample temperatures starting around 1300 K. The Pu sample for the efficiency measurement contained 40 ± 0.4 $\upmu$l of the 100 ppm $^{242}$Pu solution, dried on a 0.001 inch thick Zr foil (approximately 6x8 mm in size) and then wrapped in the foil. To verify the concentration of the 100 ppm master solution, three samples of 1 $\upmu$l of the 100 ppm solution were measured by gross alpha counting using a Canberra Series 5 XLB low background counting system with an efficiency calibrated with a NIST traceable standard. The average activity measured with the three samples was 1020.15 ± 13.37 dpm, very close to the expected activity of 1046.5 ± 10.8 dpm per $\upmu$l. It is worth noting that the shorter lived Pu isotopes with high specific activity account for 16% of the alpha activity even when present at trace levels.

## Results

### Intermediate excited states

We scanned $\lambda _2$ over the range of 767–850 nm with $\lambda _1$and $\lambda _3$ fixed. The scan was conducted two times with $\lambda _3$ = 761.95 and 763.07 nm, respectively, which were chosen arbitrarily such that the combined energy of the three photons exceeded the ionization energy of the atom. The intermediate states observed in the first scan with $\lambda _3$ = 761.95 nm were confirmed in the second scan ($\lambda _3$ = 763.07 nm). Eighteen resonance lines were observed, corresponding to resonant transitions from the first excited state to intermediate excited states of even-parity in the energy range 35568–36701 ${\text {cm}}^{-1}$. The line centroid and the full width at half maximum (FWHM) of the spectral lines were obtained by fitting with Gaussian profiles. The results are presented in Table 1. Column 2 is the average line centroid of several scans, using both increasing and decreasing photon energy, for each resonance. The uncertainty of the centroid position includes the standard deviation of the measurements and the 0.02 ${\text {cm}}^{-1}$ systematic error, added in quadrature. Column 3 is the centroid laser wavelength. Column 4 is the intermediate level energy E$_2$ obtained by adding the energy of the first excited state, 23765.98 ± 0.02 ${\text {cm}}^{-1}$ for $^{242}$Pu^26^, to the line centroid. The total angular momentum J of these intermediate states of even-parity could be 0, 1, and 2. Five of the observed intermediate states could match with the previously known states with J = 2 or 0. Columns 5 and 6 give the level energy of these known states in $^{242}$Pu from the corresponding energy for $^{240}$Pu and the isotope shifts of $^{240-242}$Pu^36^ and their J value, respectively. As shown, the measured energy positions agree well with the literature values. The other intermediate levels have not been previously documented. The average FWHM of these resonances was 0.25 ± 0.06 ${\text {cm}}^{-1}$, which was larger than the Doppler broadening (approximately 1.5 GHz) and limited by the bandwidths of the lasers.

**Table 1:** Measured resonance centroids and energy positions of the intermediate excited states of even-parity observed in $\lambda _2$ scans.

Peak #	Centroid (${\text {cm}}^{-1}$)	$\lambda _2$ (nm)	E$_2$ (${\text {cm}}^{-1}$)	Refs.^25,35^	
				E (${\text {cm}}^{-1}$)	J
1*	11802.75(2)	847.26	35568.73(3)	35568.70(3)	2
2*	12024.67(4)	831.62	35790.65(4)		
3	12160.72(4)	822.32	35926.70(4)		
4*	12211.30(4)	818.91	35977.28(4)		
5	12264.24(4)	815.38	36030.22(4)		
6	12371.68(4)	808.30	36137.66(4)	36137.60	2
7	12455.62(4)	802.85	36221.60(4)		
8*	12547.64(4)	796.96	36313.62(4)		
9	12570.12(4)	795.54	36336.10(4)		
10	12577.22(2)	795.09	36343.20(3)	36343.05	0
11	12602.38(5)	793.50	36368.36(5)		
12	12685.63(3)	788.29	36451.61(3)		
13*	12686.36(3)	788.25	36452.34(3)		
14	12736.96(3)	785.12	36502.94(3)	36502.88	2
15	12790.12(3)	781.85	36556.10(4)		
16	12843.84(3)	778.58	36609.82(4)	36609.74	2
17*	12923.51(3)	773.78	36689.49(3)		
18*	12934.51(3)	773.13	36700.50(3)		

The last two columns are the energy and J values of the known levels for $^{242}$Pu from direct measurement^26^ and the $^{240-242}$Pu isotope shifts^36^.

Seven $\lambda _2$ resonances that were clearly visible in the first scan with $\lambda _3$ = 761.95 nm were chosen as SES for further investigation of three-step schemes, as marked with (*).

Two of the intermediate levels, E$_2$ = 35568.73 ± 0.03 ${\text {cm}}^{-1}$ ($\lambda _2$ = 847.26 nm) and 36137.66 ± 0.04 ${\text {cm}}^{-1}$ ($\lambda _2$ = 808.3 nm), have been previously used as the SES in three-step resonant ionization of Pu^17,27,29,30,44^. We selected seven of the relatively strong $\lambda _2$ resonances as SES for further investigation of three-step schemes, as marked with (*) in column 1 of Table 1.

### Three-step resonant ionization

**Figure 3:**
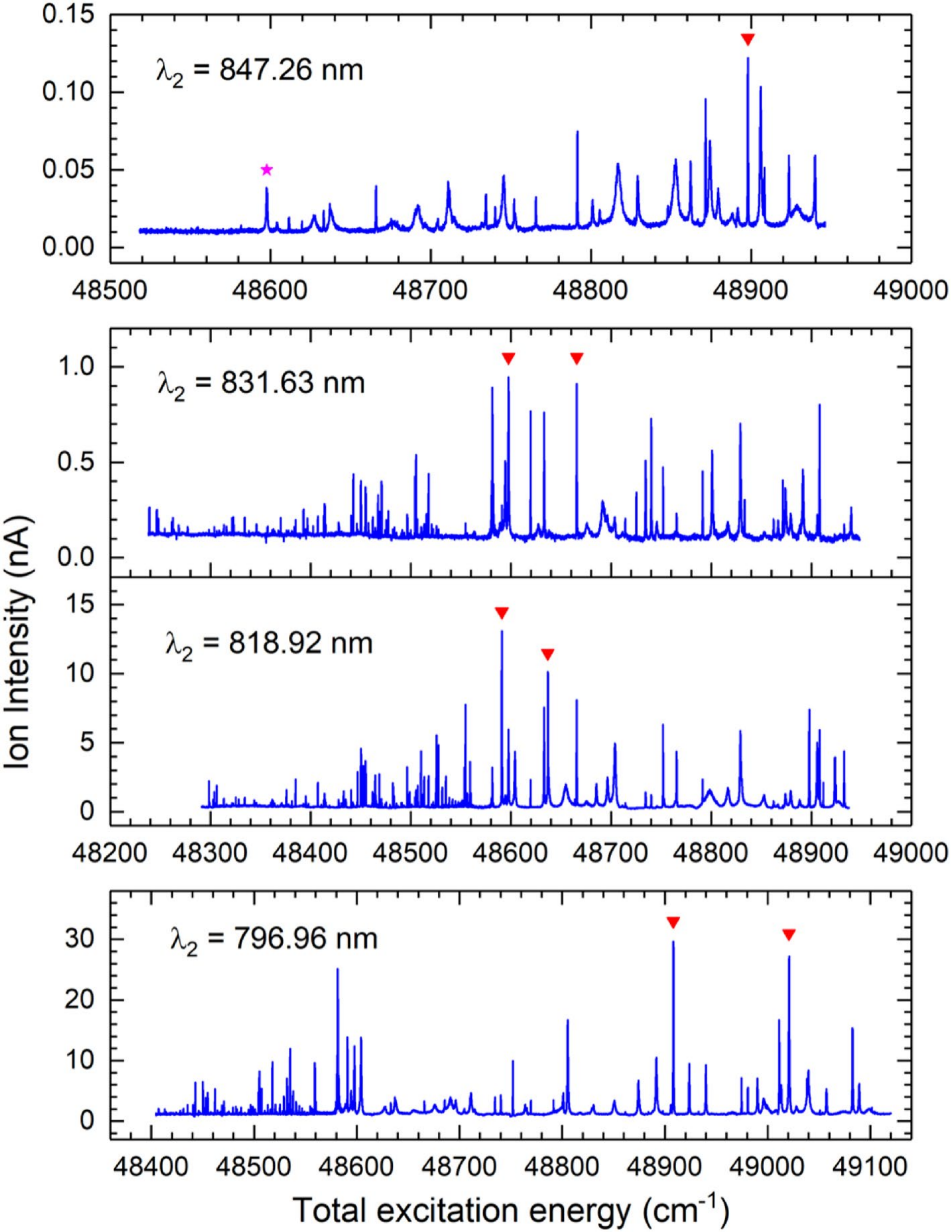
Three-step photoionization spectra excited from four selected SES (#1–#4). Inverted red triangle are selected lines for further evaluation. In the top plot, red star is the line at $\lambda _3$ = 767.53 nm.

**Figure 4:**
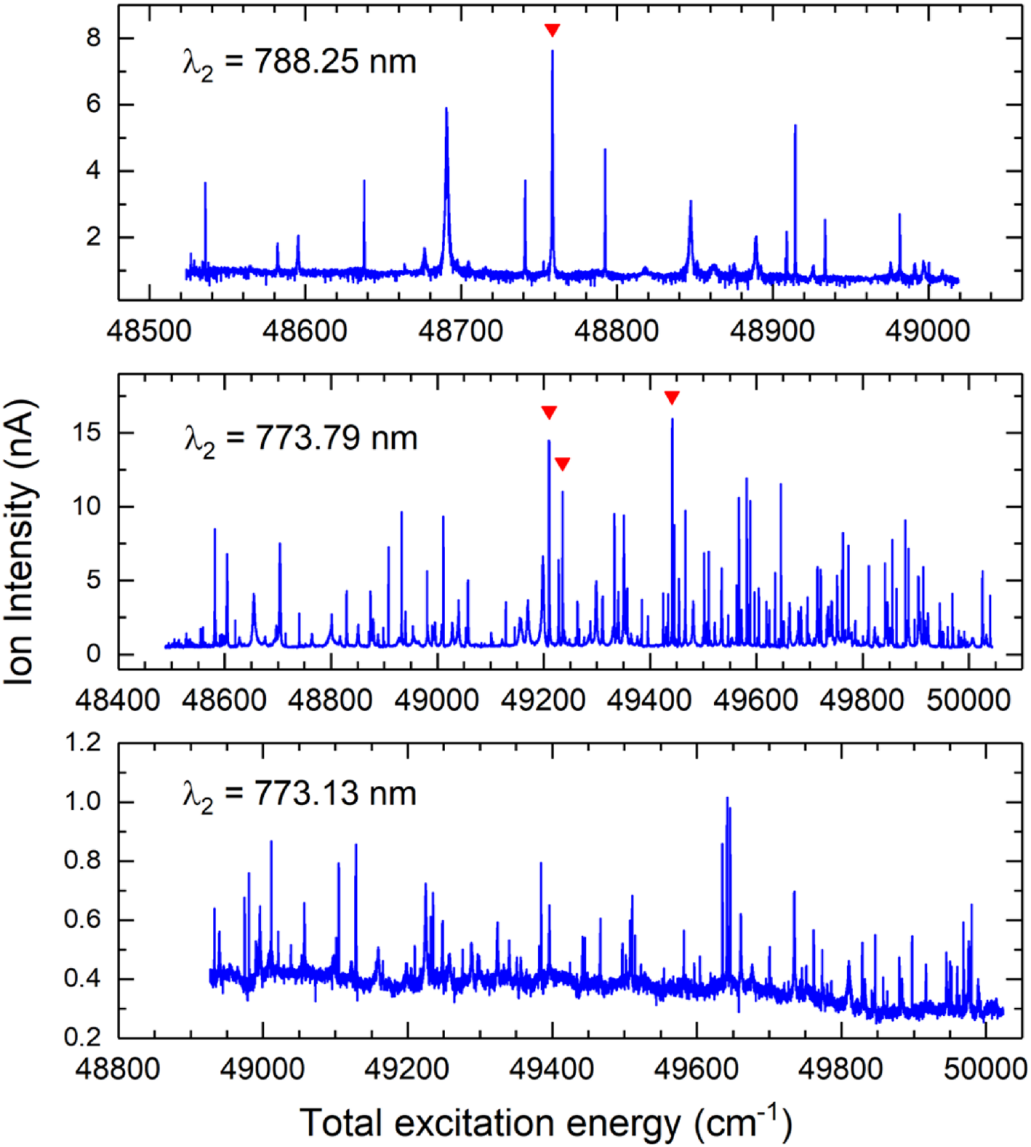
Three-step photoionization spectra excited from selected SES #5–#7. Inverted red triangle are selected lines for further evaluation.

Three-step photoionization spectra were obtained by scanning the third laser wavelength $\lambda _3$, while the first and second lasers were tuned to the respective transitions. The results are presented in Figs. 3 and 4 as a function of the total excitation energy obtained by adding the third photon energy to the level energy of the SES. Hundreds of resonance lines were observed from different intermediate states. The spectral line centroids are determined by fitting the resonance peaks to Gaussian or Fano profiles^45^. Each spectrum was measured 2–3 times by scanning $\lambda _3$ with increasing and decreasing photon energies. The final level energies are given by the average values of the scan-up and scan-down measurements. Hundreds of high-lying excited states and autoionizing states of odd-parity in the energy range of 48238–49510 ${\text {cm}}^{-1}$ were observed.

As noted, the ion intensities from different SESs are significantly different. This results from the fact that the measurements were conducted at different times and under different ion source operation conditions. It may also reflect the overall ionization efficiencies of the different excitation schemes. Therefore, in order to identify the most efficient scheme, we needed to compare the schemes under similar experimental conditions and with the lasers optimized for the respective schemes. For this purpose, we selected the strongest lines in the spectra from different SESs for further evaluation. A total of eleven resonances were chosen, as marked in Figs. 3 and 4. Table 2 lists the corresponding second and third excitations as well as the total excitation energy of the three-step schemes. Nine of the selected resonances corresponded to ionization via AI states above the IP. No resonance was selected for the SES at $\lambda _2$ = 773.13 nm for the following reasons. First, it yielded small ion currents with significant non-resonant contribution. As a results, the relative resonance enhancement was smaller than other SESs. Second, we also observed large fluctuations in the ion current, mostly due to the non-resonant contribution. Such fluctuations were not observed with other SESs and not desired for the intended applications. It is noted that the relative ion yields depend not only on the ionization schemes but also on other factors such as the laser setup, laser-neutral overlapping and interaction, ion transportation and detection, etc.

**Table 2:** Comparison of the strong resonances from selected SESs with relative ion intensity normalized to the first scheme.

SES (${\text {cm}}^{-1}$)	$\lambda _2$ (nm)	Step 3 (${\text {cm}}^{-1}$)	$\lambda _3$ (nm)	Total E (${\text {cm}}^{-1}$)	Rel. int.
35568.73(3)	847.26	13329.10(3)	750.24	48897.83(4)	1
35790.65(4)	831.62	12806.93(2)	780.83	48597.58(5)	1.2
	831.62	12875.04(2)	776.70	48665.68(5)	1.1
35977.28(4)	818.91	12613.62(2)	792.79	48590.90(5)	4.5
	818.91	12659.64(2)	789.91	48636.92(5)	4.0
36313.62(4)	796.96	12594.47(2)	794.00	48908.07(5)	4.2
	796.96	12707.15(2)	786.96	49020.77(5)	4.1
36452.34(3)	788.25	12306.02(2)	812.61	48758.29(6)	4.1
36689.49(3)	773.78	12751.88(2)	784.20	49441.36(4)	2.6
	773.78	12520.34(2)	798.70	49209.82(4)	2.5
	773.78	12545.75(2)	797.08	49235.23(4)	2.1

The first excitation was the same for all the schemes (Fig. 2).

Two of the previously known three-step ionization schemes for Pu trace analysis are marked in the top spectrum from SES #1 ($\lambda _2$ = 847.26 nm) in Fig. 3 as they are only different in the 3rd-step excitation: (inverted red triangle) $\lambda _3$ = 750.24 nm and (red star) $\lambda _3$ = 767.53 nm. The former was used by Raeder et al.^17^ to demonstrate an overall detection limit of 10$^4$ - 10$^5$ Pu atoms by in-source RIMS. It was the strongest resonance from SES #1 observed in this work. However, the latter was a relatively small resonance in the spectrum.

The selected three-step schemes were evaluated using the procedure very similar to our previous work^10,16^. Briefly, the ion source was maintained at a heating current of 300 A, where the Pu sample temperature was estimated to be on the order of 1600 K. The lasers were first tuned to a reference ionization scheme, optimized in wavelength and power, and the mass-selected $^{242}$Pu ion intensity was recorded. Then, the second and third lasers were quickly tuned to another scheme, optimized, and the corresponding ion current was measured, and so on. Periodically, the lasers were tuned back to the reference scheme to account for possible variations in the evaporation rate of the Pu sample and drifts in the ion source condition and beamline tuning. The reference scheme was the known scheme with $\lambda _2$ = 847.26 nm and $\lambda _3$ = 750.24 nm^17^. The measured ion intensities obtained with other schemes were normalized to this reference scheme and the resulting relative intensities are given in Column 6 of Table 2.

All the selected schemes were found to produce more ion currents than the reference scheme. Five schemes from three different SESs showed similar relative intensities of 4 - 4.5. The scheme that gave the largest relative ion intensity of 4.5 was $\lambda _2$ = 818.91 nm and $\lambda _3$ = 792.79 nm, which excited the Pu atoms to a high-lying excited state at 48590.90 ± 0.05 ${\text {cm}}^{-1}$ below the continuum followed by ionization by infrared radiation, electric field, collisions, etc., with the exact mechanism(s) not identified.

### Ionization efficiency

The overall ionization efficiency for Pu was measured using the three-step scheme that gave the largest relative ion intensity in this study, that is,

$$\begin{aligned} 7s^2 \; {^7F}_0 \xrightarrow {\lambda _1} 7s7p ^7D_1^o \xrightarrow {\lambda _2} 35977.28 \; \pm \; 0.04 \; {\mathrm {cm}}^{-1} \xrightarrow {\lambda _3} 48590.90 \; \pm 0.05 \; {\mathrm {cm}}^{-1}, \end{aligned}$$

where $\lambda _1$ = 402.77 nm, $\lambda _2$ = 818.91 nm, and $\lambda _3$ = 792.79 nm. The measurement procedure was as described below and the three Ti:Sapphire lasers were tuned and optimized to these three wavelengths. A quantified sample with a known number of $^{242}$Pu atoms was gradually heated in the ion source while the $^{242}$Pu ion beam intensity extracted from the ion source and mass-selected was continuously measured until the sample was completely evaporated out of the ion source. The ionization efficiency was then given by the ratio of the integrated total number of detected ions to the total number of neutral atoms initially in the sample.

**Figure 5:**
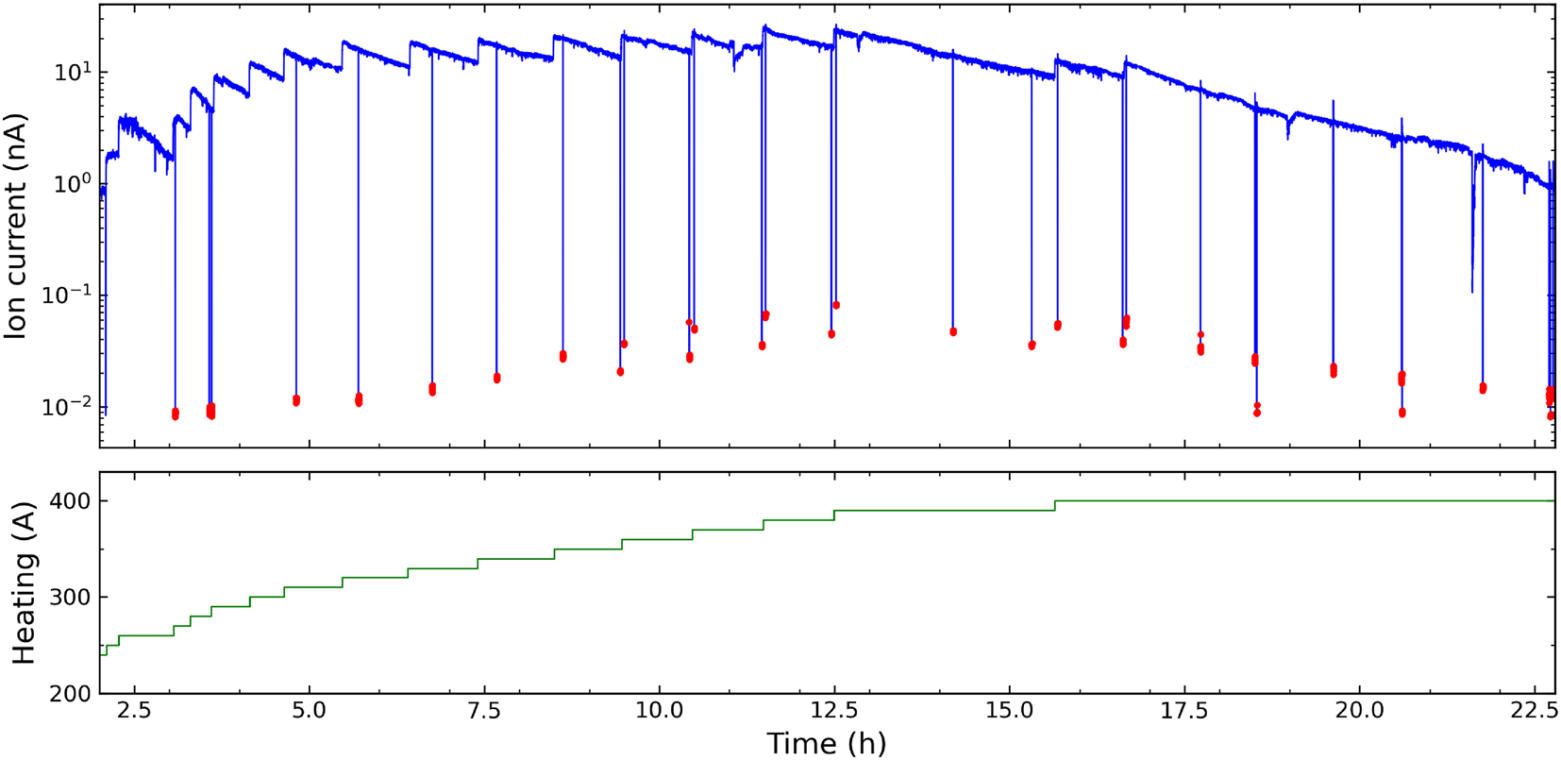
Time evolution of the efficiency measurement with a sample of $9.97 \times 10^{15}$ atoms of $^{242}$Pu. The blue line shows the $^{242}$Pu ion current measured. The sample was gradually heated and the ion signal was recorded as a function of time. The surface ionization (non-resonant background) was measured by blocking the lasers (red points). The green line shows the heating current from 210 to 400 A. The corresponding cavity temperature was estimated in the range of 1435–2200 K.

Ions of $^{242}$Pu ions were observed starting at a heating current of 220 A, at which the estimated cavity temperature was T $\sim$ 1490 K and the Pu sample was at T $\sim$ 1300 K. At the maximum heating current 400 A, the cavity temperature was on the order of 2200 K. Figure 5 shows the $^{242}$Pu ion beam intensity recorded as a function of time during the efficiency measurement. The step increases in the ion intensity correspond to stepwise increases of the electrical heating current in 10-A steps. The peak $^{242}$Pu$^+$ intensity was about 25 nA at heating current of 370–380 A. Increasing the heating current to 390 A and 400 A, between t = 16 to 17 h, only resulted in smaller increases followed by faster decreases in the ion intensity. This indicated that the sample was close to depletion. The measurement continued until the ion intensity dropped to about 0.9 nA while the ion source was heated at 400 A. During the measurement, the ion intensity from surface ionization was frequently checked by blocking all the laser beams. This corresponds to the vertical drops of the ion intensity down to the baseline in Fig. 4. The maximum surface ion intensity observed was on the order of 80 pA; an average of 0.35 ± 0.22% of the ions were from surface ionization. Based on weight we estimate that the 40 $\upmu$l sample contained a total 9.97 ± 0.1 x 10$^{15}$ $^{242}$Pu atoms which is consistent with the estimate based on the measured activities, of 9.72 ± 0.16 x10$^{15}$ $^{242}$Pu. As a conservative estimation, the total number of $^{242}$Pu atoms is 9.97 ± 0.25 x 10$^{15}$ with a larger uncertainty taking into account of the difference between the estimations by weight and by measured activities. The measured ion current, after subtracting the surface ions, gave an overall ionization efficiency of 51.1 ± 1.3 %.

### Saturation curves

**Figure 6:**
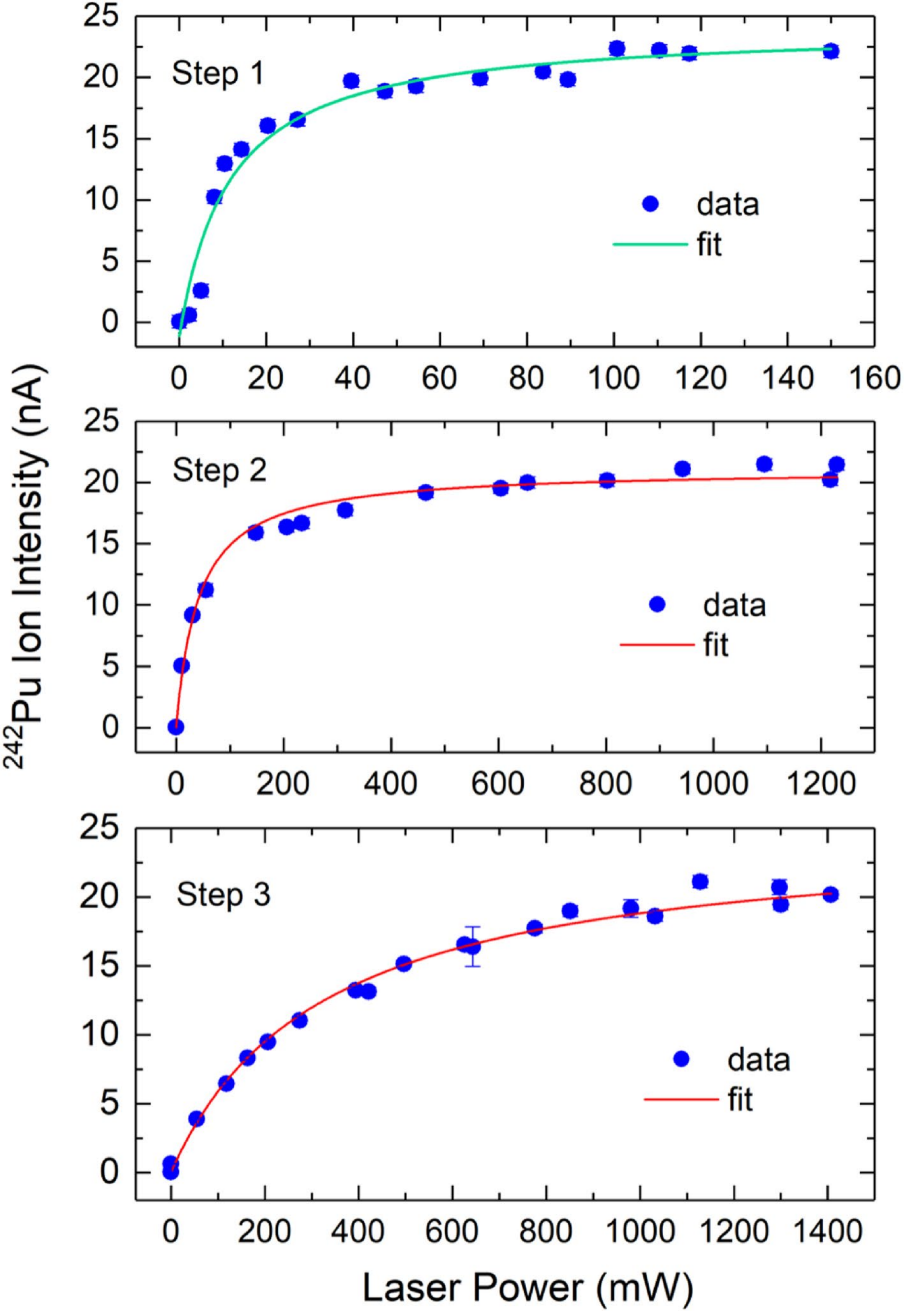
Measured saturation curves for step 1 ($\lambda _1$ = 402.77 nm), step 2 ($\lambda _2$ = 818.92 nm), and step 3 ($\lambda _3$ = 792.79 nm) excitations. The error bars represent one standard deviation of the measurement uncertainty.

The laser output power for the efficiency measurement was about 100 mW, 1.5 W, and 1.6 W for the first, second, and third steps, respectively. The saturation behavior of the three excitation steps used for the efficiency measurement have been studied. Figure 6 shows the measured $^{242}$Pu ion intensity as a function of the laser output power for each excitation, where the solid lines represent the saturation curves obtained by fitting the experimental data to the following saturation equation

1 $$\begin{aligned} I(P) = I_0 + A \frac{P/P_{sat}}{1 + P/P_{sat}} \end{aligned}$$

where I is the $^{242}$Pu ion intensity, P is the laser power, P_sat_ is the saturation power, $I_0$ is the background ion intensity due to non-laser ionization, and A is a constant. The extracted saturation power is P$_{sat}$ = 12 ± 2 mW, 45 ± 5 mW, and 325 ± 67 mW for the first, second, and third excitations, respectively. Thus, all three transitions were well saturated in the efficiency measurement. The selectivity of the three-step scheme was also checked by blocking the individual lasers and we found that approximately 97% of the laser ionized ions were produced by three-step, three-photon ionization and 3% by $\lambda _1$ + $\lambda _2$. It should be pointed out that the laser saturation power was measured in the laser room after the lasers beams were merged. From that point, about 70–80% of the laser power was injected into the hot-cavity. That is, the actual saturation power for the excitation steps in the interaction region was about 70–80% of the above fitted P_sat_ values.

## Discussion

We have demonstrated an ionization efficiency of 51.1 ± 1.3 % for Pu with the best three-step resonance ionization scheme identified. It should be noted that several other schemes with relative intensity of $\ge$ 4 in Table 2 could be similarly efficient. Our result is a factor of 10 improvement over the previously reported efficiency^17^ using the reference scheme given in Table 2 ($\lambda _1$ = 402.77 nm, $\lambda _2$ = 847.26 nm, and $\lambda _3$ = 750.24 nm). The overall ionization efficiency depends not only on the ionization scheme but also on other factors including sample evaporation, laser-atom overlapping, and transmission of the ion optics, etc. The efficiency of the reference scheme obtained by Raeder et al. could be limited by the performance of their apparatus. In comparison, the hot-cavity RILIS in the present study has shown to give higher overall efficiencies than similar laser ion sources due to its longer laser-atom overlapping region and near 100% ion transmission to the FC detector. Raeder et al.^17^ reported a detection limit of 10$^4$ - 10$^5$ Pu atoms by in-source RIMS using the reference scheme.

The ground state of Pu (Fig. 2) has several fine structure levels with substantial thermal populations at high temperatures. The selected three-step scheme can only access the $^{242}$Pu atoms in the ground state 5f$^6$7s$^2$ $^7$F$_0$ at 0 ${\text {cm}}^{-1}$. During the efficiency measurement, the ion source was gradually heated to a heating current of 400 A. The average temperature of the Pu sample was estimated to be on the order of 1800 K. At this temperature, about 55% of the Pu atoms are in the ground state $^7F_0$, with 28% and 9% in the $^7F_1$ level at 2203.606 ${\text {cm}}^{-1}$ and the $^7F_2$ level at 4299.659 ${\text {cm}}^{-1}$, respectively. It is possible to further increase the overall ionization efficiency by adding another laser that can provide $\lambda _1$ = 463.77 nm photons to excite the thermal population in the $^7F_1$ level to the same excited state 5f$^6$7s7p $^7$D$_1^o$. Such enhancement has been demonstrated to increase the laser ionization yield for $^{26}$Al^46^ and U^37^.

Our study is limited to three-step ionization schemes. Two-step resonance ionization schemes have been used for Pu and other actinides^47–50^. They are desired since only two lasers are needed. However, they use frequency-doubled photons for both steps. Studying two-step photoionization spectra requires an autotracking system for continuous scan of frequency-doubled light, which is not available for the present work.

## Conclusion

Three-step resonance ionization spectroscopy on $^{242}$Pu has been studied using Ti:Sapphire lasers and a hot-cavity laser ion source. We observed eighteen intermediate excited states of even parity in the energy range 35568–36701 ${\text {cm}}^{-1}$, thirteen of them were newly observed. Three-step photoionization spectra from seven intermediate states were measured. The strongest resonance ionization transitions from different SESs were selected and evaluated under similar ion source operating conditions. This led to the identification of several highly efficient three-step resonant ionization schemes for Pu. The scheme that yielded the largest relative ion intensity was selected for an efficiency measurement and an overall ionization efficiency of 51.1 ± 1.3% was obtained. To our knowledge, this is the highest laser ionization efficiency for plutonium and is an order of magnitude improvement over the previously reported laser ionization efficiency for Pu. This high efficiency could significantly improve RIMS analysis of ultra-trace Pu isotopes.
